# Supplementary material for: The Molecular Logic of Gtr1/2 and Pib2 Dependent TORC1 Regulation in Budding Yeast
Source: bioRxiv. 2023 Dec 7:2023.12.06.570342. Preprint. [Version 1] doi: 10.1101/2023.12.06.570342 (PMC10723367; doi:10.1101/2023.12.06.570342)
Supplement: Supplementary file 1 [file NIHPP2023.12.06.570342V1-supplement-1.pdf]

bioRxiv preprint doi: https://doi.org/10.1101/2023.12.06.570342; this version posted December 7, 2023. The copyright holder for this preprint (which was not certified by peer review) is the author/funder, who has granted bioRxiv a license to display the preprint in perpetuity. It is made available under aCC-BY 4.0 International license.

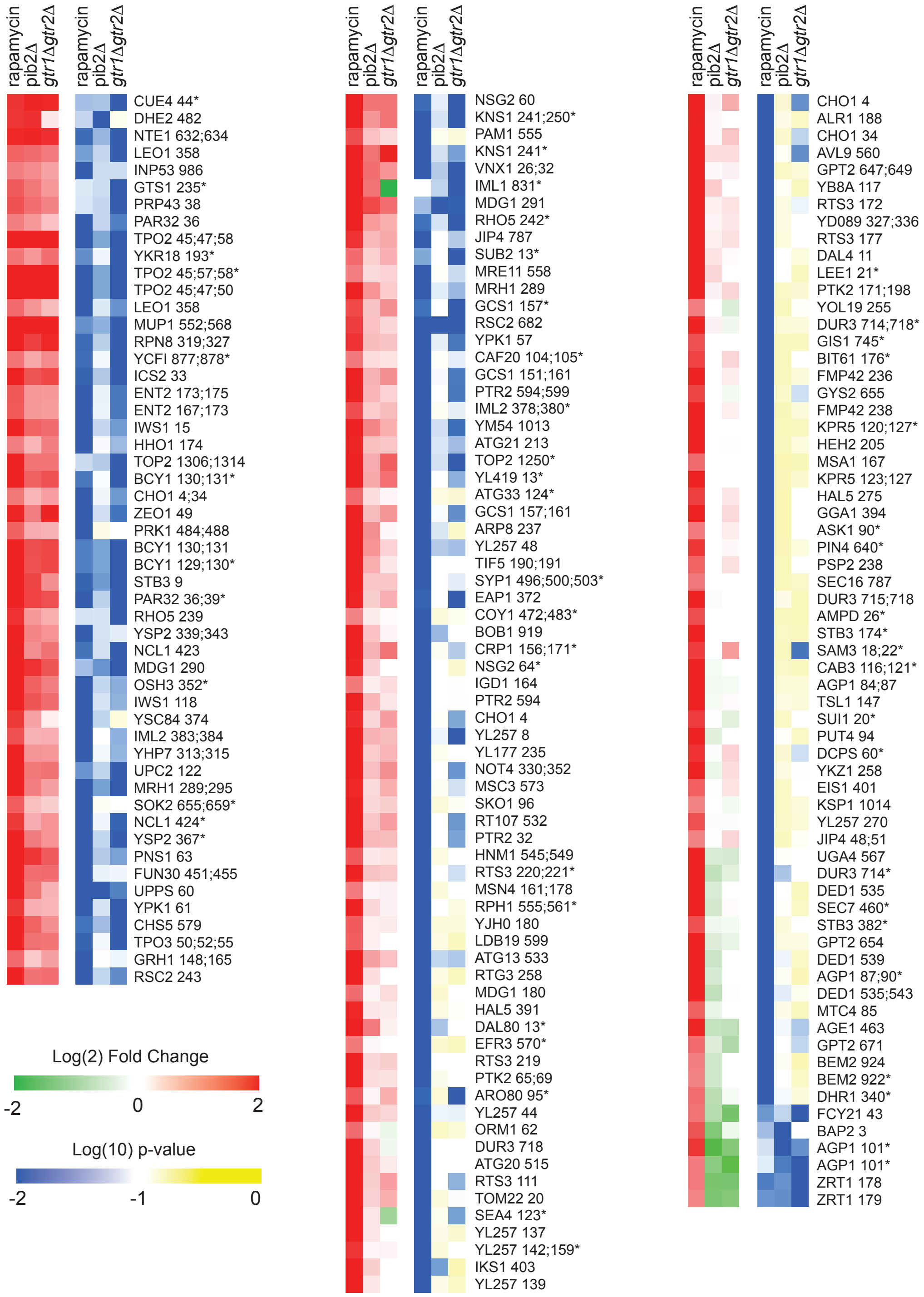

Figure 4 S1. Phosphopeptides significantly upregulated in rapamycin. Heatmap showing the abundance of all the phosphopeptides that are upregulated in rapamycin (>2-fold with p-value <0.01). The figure is laid out as described for Fig. 4.

bioRxiv preprint doi: https://doi.org/10.1101/2023.12.06.570342; this version posted December 7, 2023. The copyright holder for this preprint (which was not certified by peer review) is the author/funder, who has granted bioRxiv a license to display the preprint in perpetuity. It is made available under aCC-BY 4.0 International license.

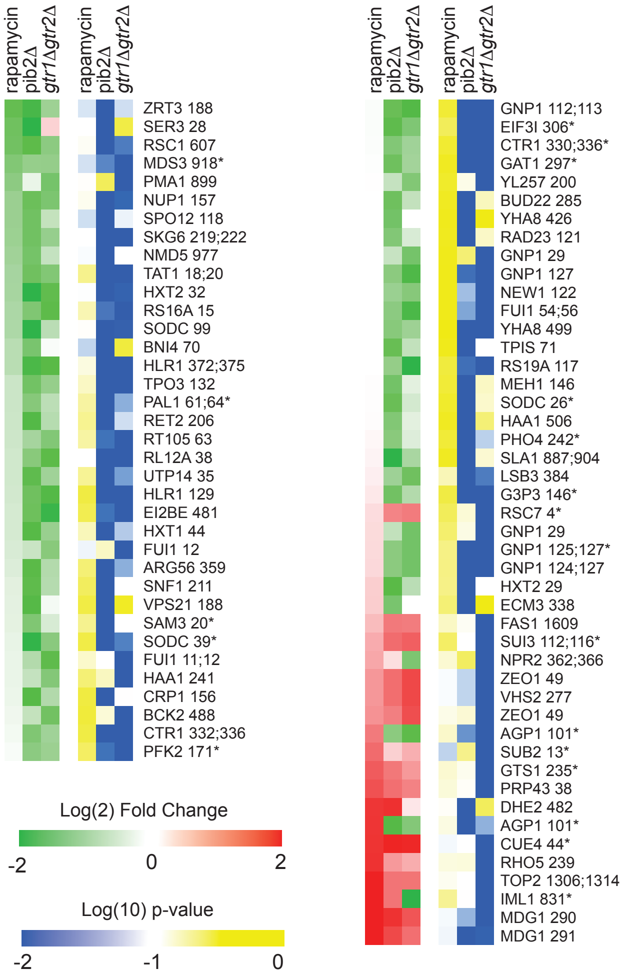

Figure 4 S2: Phosphopeptides significantly up/downregulated by deletion Gtr1/2 or Pib2 but not by rapamycin. Heatmap showing the abundance of the phosphopeptides that are up or down regulated in the Gtr1/2 and/or Pib2 delete strains (>2-fold with p-value <0.01) but with no statistically significant change in rapamycin treatment (i.e., not assigned to Fig. 4, or 4S1). The figure is laid out as described for Fig. 4. Note that a significant number of the peptides in this heatmap are sensitive to rapamycin but failed to make the (2-fold and p<0.01) cutoff threshold due to noise (see top left column, and bottom right column). Some of the other data in this heatmap may also represent noise—particularly for the peptides where there is a significant change in the *pib2*Δ or *gtr1*Δ*gtr2*Δ strains, but not both. Where there is a *bona fide* change in the *pib2*Δ and *gtr1*Δ*gtr2*Δ strains but no change in rapamycin, for example at the multiple target sites in the glutamine transporter Gnp1 (top and middle, right column), we suspect that there are

bioRxiv preprint doi: https://doi.org/10.1101/2023.12.06.570342; this version posted December 7, 2023. The copyright holder for this preprint (which was not certified by peer review) is the author/funder, who has granted bioRxiv a license to display the preprint in perpetuity. It is made available under aCC-BY 4.0 International license.

competing TORC1 dependent regulatory events. The first is a Gtr1/2 and Pib2 sensitive phosphorylation event. The second is a TORC1 repressed (Gtr1/2 and Pib2 insensitive) phosphorylation event. This dual control mechanism could be used to activate/repress proteins during intermediate, but not complete, starvation.

bioRxiv preprint doi: https://doi.org/10.1101/2023.12.06.570342; this version posted December 7, 2023. The copyright holder for this preprint (which was not certified by peer review) is the author/funder, who has granted bioRxiv a license to display the preprint in perpetuity. It is made available under aCC-BY 4.0 International license.

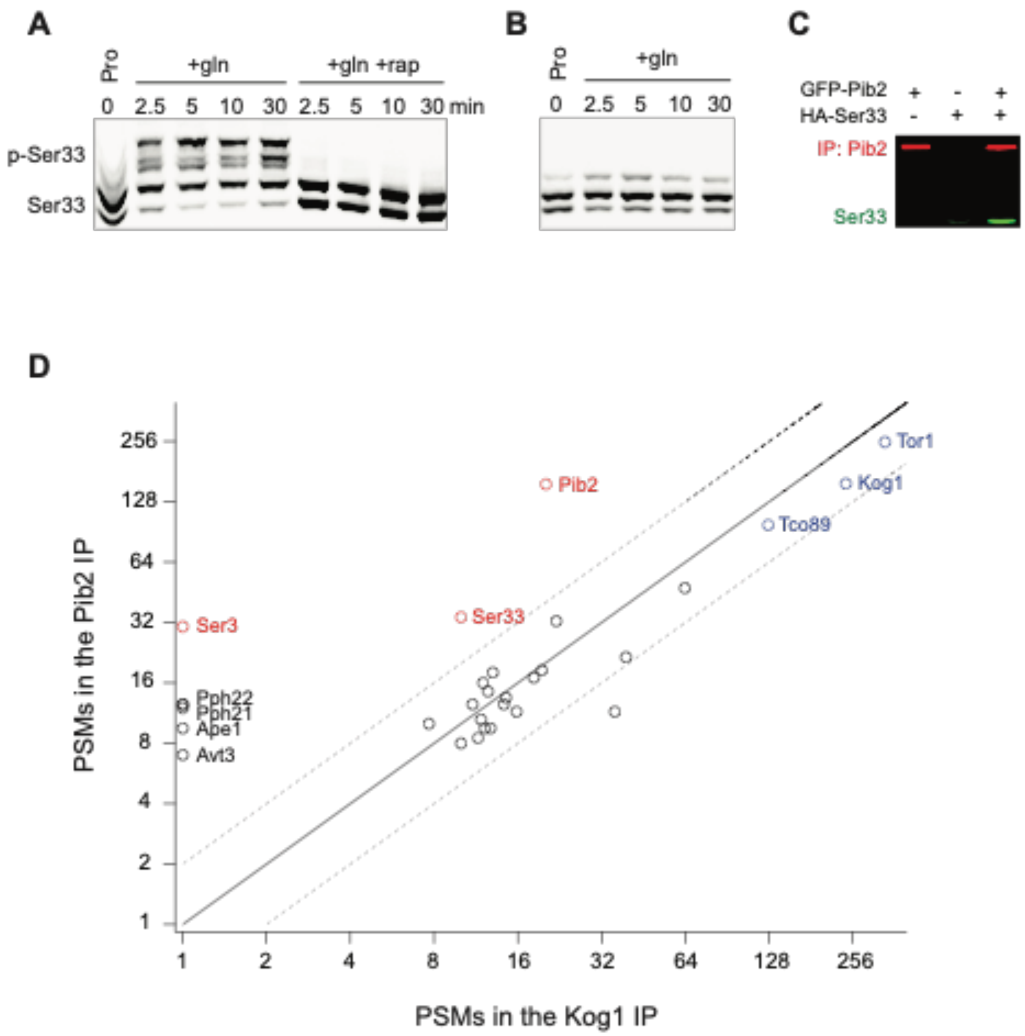

Figure 5 S1. Rapid Ser33 phosphorylation by TORC1 in response to glutamine stimulation and evidence for a direct interaction with Pib2. (A) Phos-tag gel examining Ser33 phosphorylation in cells grown to mid-log phase in proline medium (time 0) and then after addition of 0.5 g/L glutamine (with or without rapamycin). **(B)** Phos-tag gel examining the phosphorylation of SER33^{S7A} grown to mid-log phase in proline medium (time 0) and then after addition of 0.5 g/L glutamine. **(C)** Co-immunoprecipitation showing an interaction between GFP-Pib2 and Ser33-HA during log phase growth (repeat of Fig. 5f). **(D)** Comparison between the amount of 30 proteins captured (as determined by the number of Peptide Spectral Maps) in a Pib2 immunopurification and a Kog1 immunopurification. Proteins are included on the graph if they were captured in both published Pib2 IPs [73] (many proteins are captured in a Kog1 IP, but not a Pib2 IP).
